# Genome-wide investigation of mRNA lifetime determinants in *Escherichia coli* cells cultured at different growth rates

**DOI:** 10.1186/s12864-015-1482-8

**Published:** 2015-04-09

**Authors:** Thomas Esquerré, Annick Moisan, Hélène Chiapello, Liisa Arike, Raivo Vilu, Christine Gaspin, Muriel Cocaign-Bousquet, Laurence Girbal

**Affiliations:** Université de Toulouse; ISBP, INSA, UPS, INP; LISBP, 135, avenue de Rangueil, 31077 Toulouse cedex 4, France; INRA, UMR792 Ingénierie des systèmes biologiques et des procédés, 31400 Toulouse, France; CNRS, UMR5504, 31400 Toulouse, France; Laboratoire de Microbiologie et Génétique Moléculaires, UMR5100, Centre National de la Recherche Scientifique et Université Paul Sabatier, 118 Route de Narbonne, 31062 Toulouse, France; INRA, UR 875, F-31320 Castanet-Tolosan, France; Competence Center of Food and Fermentation Technologies, Akadeemia tee 15A, 12618 Tallinn, Estonia; Department of Chemistry, Tallinn University of Technology, Akadeemia tee 15, 12618 Tallinn, Estonia

**Keywords:** mRNA decay, Determinants, Growth rate, Genome-wide analysis, *Escherichia coli*

## Abstract

**Background:**

Changes to mRNA lifetime adjust mRNA concentration, facilitating the adaptation of growth rate to changes in growth conditions. However, the mechanisms regulating mRNA lifetime are poorly understood at the genome-wide scale and have not been investigated in bacteria growing at different rates.

**Results:**

We used linear covariance models and the best model selected according to the Akaike information criterion to identify and rank intrinsic and extrinsic general transcript parameters correlated with mRNA lifetime, using mRNA half-life datasets for *E. coli,* obtained at four growth rates. The principal parameter correlated with mRNA stability was mRNA concentration, the mRNAs most concentrated in the cells being the least stable. However, sequence-related features (codon adaptation index (CAI), ORF length, GC content, polycistronic mRNA), gene function and essentiality also affected mRNA lifetime at all growth rates. We also identified sequence motifs within the 5′UTRs potentially related to mRNA stability. Growth rate-dependent effects were confined to particular functional categories (e.g. carbohydrate and nucleotide metabolism). Finally, mRNA stability was less strongly correlated with the amount of protein produced than mRNA concentration and CAI.

**Conclusions:**

This study provides the most complete genome-wide analysis to date of the general factors correlated with mRNA lifetime in *E. coli*. We have generalized for the entire population of transcripts or excluded determinants previously defined as regulators of stability for some particular mRNAs and identified new, unexpected general indicators. These results will pave the way for discussions of the underlying mechanisms and their interaction with the growth physiology of bacteria.

**Electronic supplementary material:**

The online version of this article (doi:10.1186/s12864-015-1482-8) contains supplementary material, which is available to authorized users.

## Background

The adaptation of cells to changing environments involves the regulation of gene expression and, in particular, of mRNA levels, through changes in the rates of mRNA synthesis and degradation. The regulation of mRNA decay was recently shown to have a significant effect on mRNA levels in response to changes in growth rate [1]. A number of molecular mechanisms regulating mRNA stability and involving mRNA-binding molecules (small RNA, protein and/or metabolite) have been described. For example, the binding of the sugar transport-related sRNA SgrS, together with Hfq and RNase E, is known to destabilize *ptsG* mRNA [2]. CsrA protein increases the amount of *flhDC* transcript present in the cell, by preventing its degradation by RNase E [3]. Riboswitches can either sequester or release RNase E cleavage sites, by triggering structural changes to the RNA following the binding of small molecules, such as lysine for the *lysC* mRNA [4]. The binding of ribosomes (ribonucleoprotein complexes) to mRNA has been reported to have a dual role, either preventing or accelerating bacterial mRNA degradation [5,6]. These studies directly highlighted the role played by mRNA sequence-related determinants, such as specific recognition sequences [7] and secondary structures [8], in the mechanisms regulating mRNA lifetime. These mechanisms have been studied and described for particular transcripts and may be specific to the transcripts concerned. However, the global mechanisms determining mRNA stability patterns relevant for the genome-wide regulation of mRNA levels have yet to be studied and characterized. The large-scale datasets already available for mRNA half-life and the development of “omics” methods have opened up new possibilities for discovering and characterizing global regulation patterns and their underlying mechanisms. However, only eight studies addressing the mechanisms determining mRNA half-life at the genome-wide scale have been published. These studies concern *Escherichia coli* [9], *Saccharomyces cerevisiae* [10], *Bacillus subtilis* [11], *Lactococcus lactis* [12,13], *Halobacterium salinarum* [14], *Bacillus cereus* [15], and *Mycobacterium tuberculosis* [16]. Significant differences in results were reported between these analyses, which may have been due to inherent biological differences between the microorganisms studied and/or differences in the methods used for data collection and statistical analysis. For example, the effect of transcript length on mRNA stability has been found to differ between microorganisms: with no effect of length observed in *S. cerevisiae*, *B. subtilis*, *B. cereus*, *H. salinarum* and *M. tuberculosis,* but a negative effect in *L. lactis* [10-16]. Two analyses in *E. coli* yielded conflicting results, with one study reporting a negative effect (longer mRNAs being less stable than shorter ones) and the other no effect of length on mRNA stability [9,17]. The effects of the various factors on mRNA stability has been systematically studied by simple pairwise linear correlation analysis, regardless of the nature of these factors, making it impossible to rank them. Furthermore, genome-wide parameters associated with mRNA lifetime have generally been investigated only for a single set of growth conditions, despite the differences in mRNA stability observed at different growth rates in *E. coli* [1], *M. tuberculosis* [16] and *L. lactis* [13]. It therefore remains unclear whether the mechanisms determining mRNA stability can change and whether they are dependent on growth rate.

We carried out a genome-wide analysis of transcript lifetimes in the model bacterium, *E. coli* MG1655, at various growth rates. We used the largest (70% of *E. coli* genes) and the most recent mRNA half-life datasets (determined by transcriptomic-based methods) available for this microorganism [1]. The half-lives ranged from 1 to 53 minutes and were shown to be dependent on growth rate [1]. However, the key factors responsible for this considerable variability of mRNA stability remain unknown. We investigated the major parameters correlated with mRNA stability, by modeling all mRNA half-lives statistically, taking into account, without the *a priori* selection of parameters, both qualitative (e.g. gene function, gene essentiality) and quantitative (e.g. mRNA concentration, codon adaptation index (CAI, a measure of synonymous codon usage bias [18])) biological factors. This made it possible to rank the factors most strongly influencing growth rate-dependent mRNA stability patterns. We found that the general parameters explained more than half the variability of mRNA stability, even in the presence of mRNA-specific regulation. The nature of the general parameters identified provides valuable insight into the genome-wide mechanisms associated with mRNA stability. For example, mRNA concentration, length and GC content are directly related to the activity of the degradation machinery; CAI and a sequence motif are related to the translational activity of ribosomes and other parameters (e.g. gene function) are related to as yet unknown mechanisms. Finally, we also investigated the role of mRNA stability regulation in determining growth rate-dependent protein levels in cells, using new proteomic datasets for *E. coli* growing at different rates.

## Results

We used a linear covariance model to identify factors relating to mRNA stability in *E. coli.* This analysis was based on large-scale mRNA half-life datasets [1], taking into account both quantitative (e.g. mRNA concentration, length, sequence composition and structure, CAI) and qualitative (e.g. gene essentiality, gene function, cellular distribution of the gene product, predicted peptide signal, polycistronic transcript) parameters (the complete list is provided in the Methods section). A previous study reported differences in the patterns of mRNA stability regulation between growth rates [1]. We therefore decided to use four linear covariance models corresponding to different growth rates (μ = 0.10, 0.20, 0.40 and 0.63 h^−1^). For each model, 1589 half-life values (corresponding to ~ 37% of all *E. coli* coding genes) were available: these datasets for mRNA half-life are the largest ever used to identify mRNA stability determinants. The most important parameters, selected on the basis of the Akaike information criterion (AIC, see the Methods section), are listed for each model in Table 1. This subset of parameters included both quantitative and qualitative parameters, reflecting intrinsic features (e.g. CAI, length, GC%, function) and extrinsic parameters dependent on the environment (e.g. [mRNA], essentiality of the gene function). The selection of these parameters resulted in a relatively good quality of fit for all four models, with determination coefficients (adjusted R^2^) of between 0.74 at μ = 0.10 h^−1^ and 0.51 at μ = 0.63 h^−1^. These results indicate that mRNA stability is well accounted for by the selected parameters, particularly at the lowest growth rates (see the Discussion).

**Table 1:** **Estimated coefficients and** ***P-*** **values of the growth rate-dependent mRNA half-life models**

**Parameter**	**Dependent variable: half-life**							
	***μ*** **= 0.10 h** ^**−1**^		***μ*** **= 0.20 h** ^**−1**^		***μ*** **= 0.40 h** ^**−1**^		***μ*** **= 0.63 h** ^**−1**^	
	**Est. Coeff.**	***P-value***	**Est. Coeff.**	***P-value***	**Est. Coeff.**	***P-value***	**Est. Coeff.**	***P-value***
**[mRNA]**	**−0.91**	*<10* ^*−226*^	**−0.80**	*1.4E10* ^*−223*^	**−0.80**	*2.9E10* ^*−225*^	**−0.79**	*4.3E10* ^*−205*^
**CAI**	**0.13**	*2.6E10* ^*−15*^	**0.18**	*1.7E10* ^*−15*^	**0.18**	*5.5E10* ^*−17*^	**0.25**	*7.9E10* ^*−28*^
**ORF length**	**−0.14**	*2.8E10* ^*−5*^	**−0.16**	*2.2E10* ^*−4*^	**−0.19**	*1.1E10* ^*−5*^	**−0.12**	*7.7E10* ^*−3*^
**5UTR + ORF length**	0.05	*9.4E10* ^*−2*^	0.06	*1.3E10* ^*−1*^	**0.14**	*9.1E10* ^*−4*^	0.09	*5.2E10* ^*−2*^
**5’UTR + ORF GC%**	**−0.05**	*4.4E10* ^*−4*^	**−0.07**	*3.1E10* ^*−4*^	**−0.10**	*1.3E10* ^*−7*^	**−0.09**	*8.0E10* ^*−6*^
**Essential genes**	**0.06**	*8.1E10* ^*−3*^	**0.12**	*4.4E10* ^*−5*^	**0.10**	*2.7E10* ^*−4*^	**0.06**	*4.5E10* ^*−2*^
**No. of genes in operon**								
1	0.29	*2.3E10* ^*−1*^	0.55	*8.1E10* ^*−2*^	0.46	*1.4E10* ^*−1*^	0.43	*2.0E10* ^*−1*^
2	**−0.15**	*2.4E10* ^*−3*^	**−0.16**	*1.1E10* ^*−2*^	**−0.29**	*3.8E10* ^*−6*^	−0.09	*1.7E10* ^*−1*^
3	**−0.16**	*1.3E10* ^*−3*^	**−0.21**	*2.0E10* ^*−3*^	**−0.31**	*2.3E10* ^*−6*^	**−0.16**	*2.1E10* ^*−2*^
4	−0.10	*8.2E10* ^*−2*^	−0.10	*1.9E10* ^*−1*^	**−0.19**	*9.0E10* ^*−3*^	−0.11	*1.5E10* ^*−1*^
5	−0.02	*7.8E10* ^*−1*^	−0.06	*4.3E10* ^*−1*^	−0.14	*8.1E10* ^*−2*^	−0.06	*4.6E10* ^*−1*^
6	**−0.14**	*3.2E10* ^*−2*^	−0.09	*3.1E10* ^*−1*^	−0.12	*1.6E10* ^*−1*^	−0.11	*2.2E10* ^*−1*^
7	0.03	*7.1E10* ^*−1*^	0.08	*4.0E10* ^*−1*^	0.06	*5.4E10* ^*−1*^	0.08	*4.3E10* ^*−1*^
8	−0.07	*6.3E10* ^*−1*^	−0.07	*6.9E10* ^*−1*^	−0.04	*8.3E10* ^*−1*^	−0.01	*9.4E10* ^*−1*^
9	−0.08	*3.9E10* ^*−1*^	0.04	*7.2E10* ^*−1*^	0.04	*7.4E10* ^*−1*^	−0.03	*8.3E10* ^*−1*^
10	−0.13	*3.1E10* ^*−1*^	−0.11	*5.2E10* ^*−1*^	−0.10	*5.1E10* ^*−1*^	0.05	*7.5E10* ^*−1*^
11	−0.29	*4.1E10* ^*−1*^	−0.52	*2.4E10* ^*−1*^	−0.43	*3.3E10* ^*−1*^	−0.65	*1.7E10* ^*−1*^
12	**0.45**	*3.3E10* ^*−4*^	**0.50**	*2.2E10* ^*−3*^	**0.74**	*4.0E10* ^*−6*^	**0.84**	*1.3E10* ^*−6*^
13	**0.48**	*1.8E10* ^*−4*^	**0.49**	*3.3E10* ^*−3*^	**0.59**	*2.9E10* ^*−4*^	0.31	*8.1E10* ^*−2*^
16	−0.12	*6.2E10* ^*−1*^	−0.36	*2.6E10* ^*−1*^	−0.28	*3.7E10* ^*−1*^	−0.48	*1.6E10* ^*−1*^
**COG annotation**								
[C]	−0.00	*1.0E10* ^*−0*^	0.02	*8.8E10* ^*−1*^	0.05	*7.5E10* ^*−1*^	−0.01	*9.3E10* ^*−1*^
[D]	**0.39**	*8.2E10* ^*−13*^	**0.53**	*5.8E10* ^*−14*^	**0.59**	*6.2E10* ^*−18*^	**0.54**	*1.3E10* ^*−13*^
[E]	−0.21	*6.5E10* ^*−2*^	−0.23	*1.3E10* ^*−1*^	−0.23	*1.2E10* ^*−1*^	**−0.41**	*1.0E10* ^*−2*^
[F]	0.01	*8.4E10* ^*−1*^	0.05	*3.1E10* ^*−1*^	0.06	*2.7E10* ^*−1*^	**0.23**	*1.8E10* ^*−5*^
[G]	−0.13	*6.6E10* ^*−2*^	−0.16	*7.5E10* ^*−2*^	−0.16	*8.4E10* ^*−2*^	**0.24**	*1.4E10* ^*−2*^
[H]	**0.12**	*2.0E10* ^*−2*^	**0.20**	*4.7E10* ^*−3*^	**0.20**	*3.6E10* ^*−3*^	0.14	*6.3E10* ^*−2*^
[I]	**−0.12**	*4.4E10* ^*−2*^	**−0.18**	*1.7E10* ^*−2*^	**−0.24**	*1.4E10* ^*−3*^	**−0.22**	*6.1E10* ^*−3*^
[J]	**0.20**	*6.2E10* ^*−3*^	**0.21**	*2.6E10* ^*−2*^	**0.23**	*1.3E10* ^*−2*^	**0.31**	*2.4E10* ^*−3*^
[K]	0.10	*7.2E10* ^*−2*^	0.04	*5.5E10* ^*−1*^	0.06	*3.8E10* ^*−1*^	−0.03	*6.8E10* ^*−1*^
[L]	−0.01	*8.4E10* ^*−1*^	−0.05	*4.4E10* ^*−1*^	−0.07	*2.7E10* ^*−1*^	−0.12	*6.6E10* ^*−2*^
[M]	**0.13**	*2.4E10* ^*−2*^	0.11	*1.6E10* ^*−1*^	0.02	*7.5E10* ^*−1*^	−0.02	*8.3E10* ^*−1*^
[N]	**−0.12**	*3.3E10* ^*−2*^	**−0.15**	*4.3E10* ^*−2*^	**−0.14**	*3.6E10* ^*−2*^	−0.11	*1.3E10* ^*−1*^
[O]	0.16	*3.0E10* ^*−1*^	0.25	*2.0E10* ^*−1*^	0.25	*1.9E10* ^*−1*^	0.28	*1.7E10* ^*−1*^
[P]	−0.10	*8.7E10* ^*−1*^	−0.12	*1.1E10* ^*−1*^	−0.11	*1.5E10* ^*−1*^	−0.13	*1.2E10* ^*−1*^
[Q]	**−0.19**	*2.4E10* ^*−3*^	**−0.25**	*2.2E10* ^*−3*^	**−0.19**	*1.5E10* ^*−2*^	−0.12	*1.5E10* ^*−1*^
[R]	−0.08	*5.4E10* ^*−1*^	−0.12	*4.6E10* ^*−1*^	−0.20	*2.2E10* ^*−1*^	−0.16	*3.5E10* ^*−1*^
[S]	0.01	*8.8E10* ^*−1*^	−0.01	*8.0E10* ^*−1*^	−0.04	*4.9E10* ^*−1*^	−0.03	*6.1E10* ^*−1*^
[T]	**−0.10**	*2.7E10* ^*−2*^	−0.12	*5.4E10* ^*−2*^	−0.10	*1.1E10* ^*−1*^	−0.12	*5.5E10* ^*−2*^
[U]	**0.15**	*4.2E10* ^*−2*^	**0.21**	*2.6E10* ^*−2*^	**0.30**	*1.3E10* ^*−3*^	0.05	*5.9E10* ^*−1*^
[V]	−0.19	*9.8E10* ^*−2*^	−0.22	*1.4E10* ^*−1*^	**−0.29**	*4.8E10* ^*−2*^	**−0.31**	*5.0E10* ^*−2*^
**Cell location**								
Cytoplasmic	−0.07	*4.0E10* ^*−1*^	−0.08	*4.3E10* ^*−1*^	N.S		N.S	
Inn. Mb. Lipo.	−0.12	*1.1E10* ^*−1*^	−0.11	*2.6E10* ^*−1*^	N.S		N.S	
Int. Mb. Pr.	−0.20	*3.9E10* ^*−1*^	−0.27	*3.8E10* ^*−1*^	N.S		N.S	
Mb. Anchored	0.04	*6.3E10* ^*−1*^	0.02	*8.6E10* ^*−1*^	N.S		N.S	
Mb. Lipo.	−0.04	*6.6E10* ^*−1*^	−0.11	*3.4E10* ^*−1*^	N.S		N.S	
Out. Mb. B-Bar Pr.	0.48	*1.4E10* ^*−1*^	0.55	*1.9E10* ^*−1*^	N.S		N.S	
Out. Mb. Lipo.	**0.34**	*3.7E10* ^*−2*^	**0.48**	*2.4E10* ^*−2*^	N.S		N.S	
Periplasmic	−0.20	*7.4E10* ^*−1*^	−0.21	*1.5E10* ^*−1*^	N.S		N.S	
Unknown	**−0.23**	*3.8E10* ^*−2*^	**−0.27**	*1.0E10* ^*−2*^	N.S		N.S	
**Peptide signal**	−0.09	*5.4E10* ^*−2*^	−0.12	*5.8E10* ^*−2*^	N.S		N.S	
**Adjusted R** ^**2**^	**0.74**		**0.56**		**0.57**		**0.51**	

The coefficients were estimated by the minimization of least squares.

[mRNA]: mRNA concentration. CAI: codon adaptation index. 5’UTR + ORF GC%: percentage of G and C bases in combined 5’UTR and ORF sequences. No. of genes in the operon: Number of genes in the operon in which the gene is located. COG annotation – [C]: Energy production and conversion, [D]: Cell cycle control, cell division, chromosome partitioning, [E] Amino acid transport and metabolism, [F]: Nucleotide transport and metabolism, [G]: Carbohydrate transport and metabolism, [H]: Coenzyme transport and metabolism, [I]: Lipid transport and metabolism, [J]: Translation, ribosomal structure and biogenesis, [K]: Transcription, [L]: Replication, recombination and repair, [M]: Cell wall/membrane/envelope biogenesis, [N]: Cell motility, [O]: Posttranslational modification, protein turnover, chaperones, [P]: Inorganic ion transport and metabolism, [Q]: Secondary metabolite biosynthesis, transport and catabolism, [R]: General function prediction only, [S]: Function unknown, [T]: Signal transduction mechanisms, [U]: Intracellular trafficking, secretion, and vesicular transport, [V]: Defense mechanisms. Cell location: Location of the gene product within the cell – Inn. Mb. Lipo.: inner membrane lipoprotein, Int. Mb. Pr.: integral membrane protein, Mb. Anchored: anchored in the membrane, Mb. Lipo.: membrane lipoprotein, Out. Mb. B-Bar Pr.: outer membrane B-barrel protein, Out. Mb. Lipo.: outer membrane lipoprotein. N.S indicates that the parameter was not selected by the AIC algorithm. Only parameters selected at least once by the AIC algorithm are shown. Model determinants (*P*-value ≤ 0.05) are displayed in bold.

We analyzed the relative contributions of the parameters and their dependence on growth rate, by considering only selected parameters with an associated *P*-value < 0.05 to be significant indicators of mRNA stability. Significant indicators of mRNA stability present in all four models were considered to be independent of growth rate; whereas a significant indicator present in at least one, but not in all the models was considered to be dependent on growth rate.

### Indicators of mRNA stability independent of growth rate

Seven significant indicators ([mRNA], CAI, ORF length, GC% in 5′UTR + ORF, number of genes in operon, function and essentiality) were identified in all the models. Their influences were quantified by estimating the coefficients of the linear covariance models (Table 1). The most influential of the quantitative parameters was mRNA concentration, in all models, with estimated coefficients below −0.79 (*P*-value < 10^−204^). The negative sign of these coefficients indicates that higher mRNA concentrations in cells are associated with a shorter half-life, whereas lower mRNA concentrations are associated with a longer half-life. The second most influential factor was CAI, which had positive estimated coefficients of between 0.13 and 0.25 (*P*-value < 10^−14^). Thus, a higher optimal codon content was associated with a longer half-life. CAI is a well known determinant of protein level in *E. coli* [19-22], as confirmed here (see below), but its effect on mRNA stability has only rarely been investigated. We found that only ORF length (no relationship was found for the length of the 5′UTR or the length of the 5′UTR + ORF) was systematically negatively correlated with mRNA half-life (estimated coefficients < −0.12, *P*-value < 10^−2^). Finally, there was a weak negative effect of the GC content of the 5′UTR + ORF on mRNA stability (estimated coefficients < −0.05, *P*-value < 10^−3^).

Transcript stability was significantly higher for essential genes [23] than for non-essential genes, for all growth rates considered (*P*-value < 0.05). Three COG categories had significant estimated coefficients for all four models, indicating an influence of gene function on mRNA half-life. The mRNAs of genes belonging to categories [D] Cell cycle control, cell division, chromosome partitioning and [J] Translation, ribosomal structure and biogenesis categories were more stable than the other mRNAs, whereas the mRNAs of genes from category [I] Lipid transport and metabolism category were less stable than the other mRNAs.

**Figure 1:**
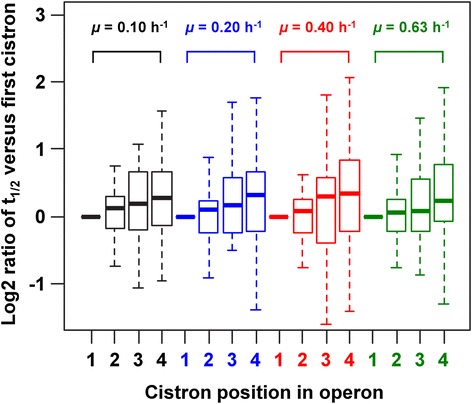
**Cistron position-dependent half-life, for polycistronic mRNAs and four growth rates.** A boxplot distribution of log2 ratios of the cistron half-lives at positions 2, 3 and 4 versus the half-life of the first cistron is shown, for the four growth rates (*μ* = 0.10, 0.20, 0.40 and 0.63 h^−1^ in black, blue, red and green, respectively). Only operons with four cistrons are represented in this figure (47 operons). This figure is representative of all the operons of other sizes. List of the 47 operons of four genes used for this figure: *appCB-yccB-appA, artPIQM, asnC-mioC-mnmG-rsmG, bcsABZC, bioBFCD, creABCD, deoCABD, ecpBCDE, fhuACDB, frdABCD, galETKM, hemCDXY, hofMNOP, hscBA-fdx-iscX, idnDOTR, iscRSUA, motAB-cheAW, mutY-yggX-mltC-nupG, nagBACD, nrdHIEF, osmF-yehYXW, pdxB-usg-truA-dedA, pepQ-yigZ-trkH-hemG, potFGHI, relA-mazEFG, rpoE-rseABC, rpoZ-spoT-trmH-recG, rpsF-priB-rpsR-rplI, rpsP-rimM-trmD-rplS, smtA-mukFEB, tatABCD, tauABCD, tgt-yajC-secDF, thrLABC, xseB-ispA-dxs-yajO, ybeZYX-lnt, ybgC-tolQRA, ybjC-nfsA-rimK-ybjN, ycaI-msbA-lpxK-ycaQ, yciSM-pyrF-yciH, yejABEF, ygfB-pepP-ubiH-visC, yheO-tusDCB, yihXY-dtd-yiiD, yjjB-dnaTC-yjjA, yqjCDEK* and *yrdD-tsaC-aroE-yrdB.*

The mRNAs of genes from operons containing three genes (estimated coefficients < −0.16, *P*-value < 0.05) were significantly less stable than those of genes belonging to operons containing 12 genes (estimated coefficients > 0.45, *P*-value < 10^−2^), suggesting that cistron stability was higher for longer operons, at all growth rates. We investigated the possible link between cistron stability and position in the operon, by comparing the stability of the first cistron with all cistrons until the last one, for all operons for which all the necessary stability data could be collected (431 of 836 operons in *E. coli*). We found that, for each growth rate, the median half-life of a cistron increased with its rank in the polycistronic mRNA. A representative example is shown, for operons with four cistrons, in Figure 1. The last cistron was, on average, 1.5 times more stable than the first cistron, in at least 62% of the operons studied (>268 operons). These data suggest that, globally, the cistrons furthest from the 5′-end of the transcript are the most stable.

**Figure 2:**
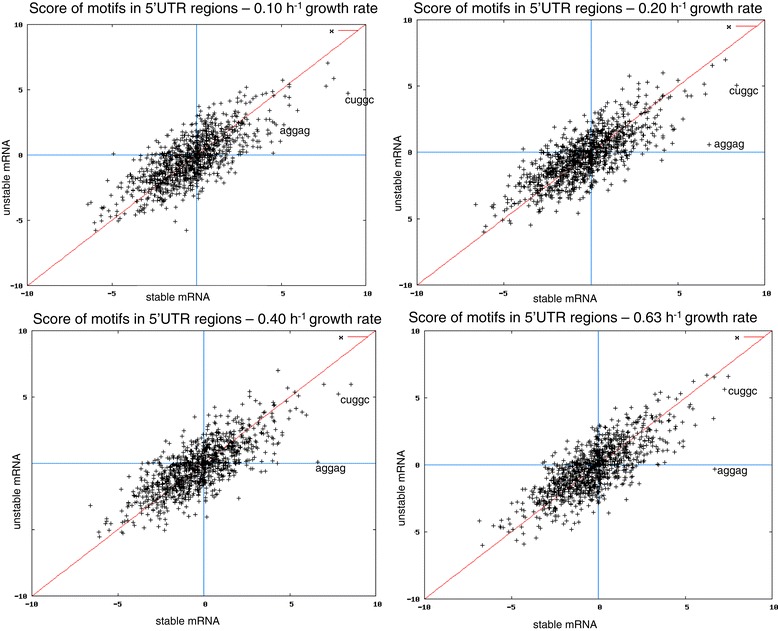
**Frequency of motifs in the 5′UTR regions of stable and unstable mRNAs.** RMES scores for all 5-nt motifs are plotted for the most stable (x-axis) and unstable mRNAs (y-axis) at the four growth rates (0.10, 0.20, 0.40 and 0.63 h^−1^, respectively). Exceptional motifs have either low (underrepresented motifs) or high (overrepresented motifs) scores. Exceptional motifs distant from the diagonal line of the scatter plot display different patterns of exceptionality in stable and unstable mRNAs.

**Figure 3:**
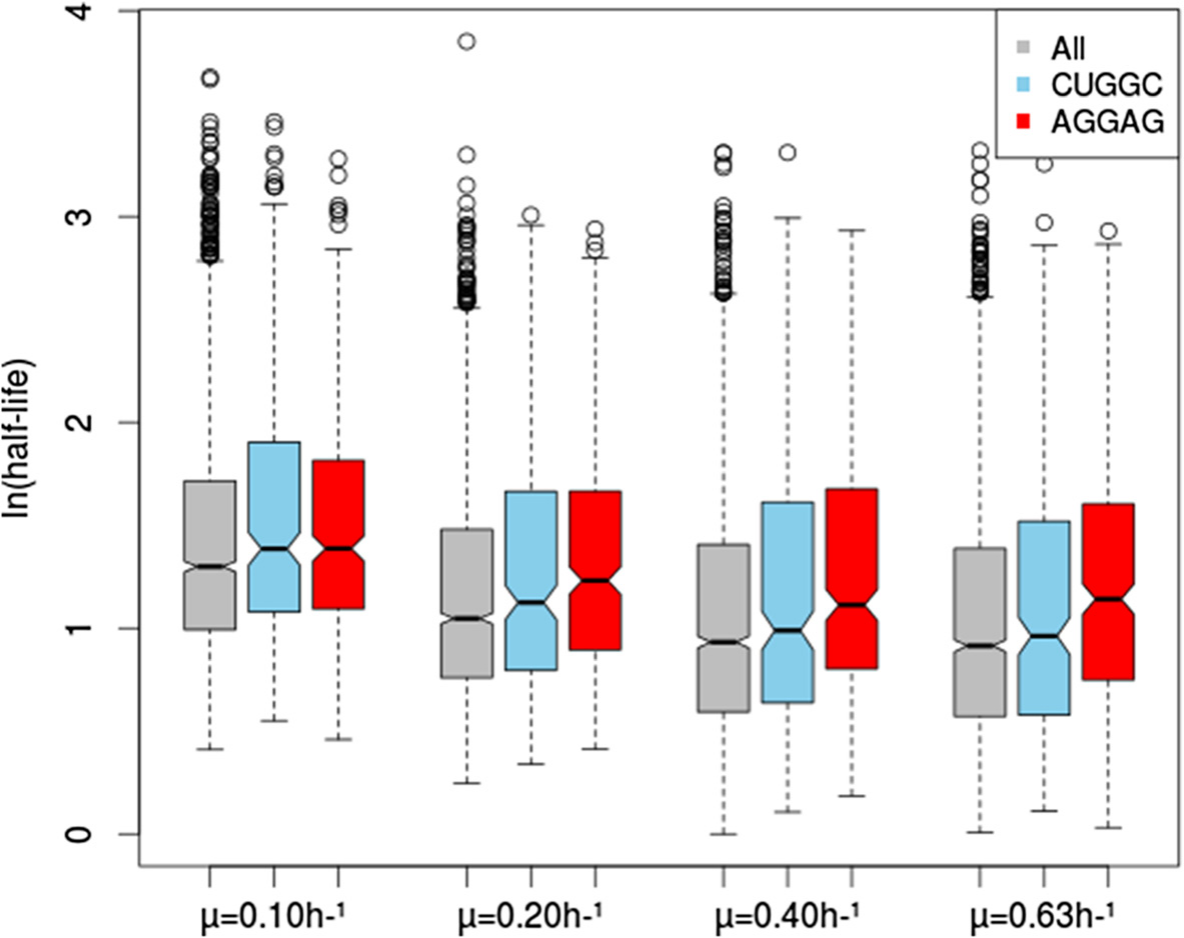
**Notched boxplot [** 45 **] of the half-lives of mRNAs with motifs in their 5′UTR.** The distribution of mRNA half-life for the entire mRNA population “All” (1937 experimentally determined 5′UTR sequences of length between 1 and 100 nucleotides) is compared with those for subpopulations of mRNAs with 5′UTRs including the “CUGGC” (blue) (#243) or “AGGAG” (red) (#319) motifs, at growth rates of *μ* = 0.10, 0.20, 0.40 and 0.63 h^−1^. The box shows the interquartile range of the data, whereas the notch indicates the 95% confidence interval around the median. If the intervals of two groups do not overlap, their medians can be assumed to be different with 95% confidence.

We also looked for sequence motifs potentially related to mRNA stability at different growth rates. We used RMES software [24] to assess the overrepresentation of all possible five-base long motifs in the experimentally demonstrated 5′UTR regions (sequence lengths between 1 and 100 nucleotides upstream from the initiation codon) of the top 25% and the bottom 25% of mRNAs ranked in terms of stability, at four growth rates (0.10, 0.20, 0.40 and 0.63 h^−1^). RMES scores of exceptionality were computed for each motif and compared between the unstable and stable mRNAs (Figure 2). The plots obtained for all the four growth rates indicated a strong correlation of motif exceptionality scores between stable and unstable mRNAs: most of the motifs were located nearby the diagonal (red) line. Nevertheless, we identified two sequence motifs overrepresented in the stable mRNAs but less or not in the unstable mRNAs. These motifs were located at the right side of the vertical blue line and at some distance from the diagonal line. At 0.20, 0.40 and 0.63 h^−1^, the AGGAG motif was found significantly more frequent in the 5′UTR of stable mRNAs than in the ones of unstable mRNAs (Figure 2). At 0.10 and 0.20 h^−1^, a second motif CUGGC was significantly overrepresented in the 5′UTR of stable mRNAs compared to the 5′UTR of unstable mRNAs (Figure 2). We validated these observations, by comparing the half-life distributions for the whole mRNA population having an experimentally demonstrated 5′UTR region and for the different subpopulations of these mRNAs including the two candidate motifs in the 5′UTR (Figure 3). The subpopulation of transcripts with the AGGAG motif in their 5′UTR was found more stable than the entire mRNA population and this was more pronounced as the growth rate increased. At the low growth rates (0.10 and 0.20 h^−1^), the subpopulation of transcripts including the CUGGC motif in the 5′UTR was globally stabilized. The AGGAG motif was mostly located in the Shine-Dalgarno region (75% of the motifs were between 2 nucleotides and 8 nucleotides upstream from the transcription start codon), with no other “hot spot” region identified in the 5′UTR, even when the motif was repeated two times. The CUGGC motif, which could be repeated as much as three times in some sequences, did not occur at specific positions and was randomly located in the 5′UTR.

The secondary structure of the 5′UTR has been reported to affect the decay of specific mRNAs by impeding access to the RNase [8,25]. Against expectations, the *Z* score of the folding energy in the −30/+24 bp region of mRNA was not identified as a general indicator of mRNA stability in any of the models. We investigated the relationship between mRNA stability and *Z* score, by comparing the stability of the mRNAs displaying the lowest *Z* scores (< −2, highest probability of stable secondary structure formation) with that of the other transcripts (Additional file 1: Figure S1). No significant difference in mRNA half-life distribution was found between the two groups. Thus, potential mRNA secondary structure in the −30/+24 bp region of the mRNA was not predictive of stability at the genome-wide level.

### Changes in mRNA stability indicators with growth rate

The significance of several parameters associated with mRNA stability was dependent on growth rate. Four COG categories were significant from μ = 0.10 to 0.40 h^−1^ but not at 0.63 h^−1^ (Table 1). By contrast, three COGs became significant only at μ = 0.63 h^−1^. At low growth rates, the mRNAs of genes belonging to categories [H] Coenzyme transport and metabolism and [U] Intracellular trafficking, secretion, and vesicular transport were more stable, whereas transcripts from categories [N] Cell motility and [Q] Secondary metabolite biosynthesis, transport and catabolism were more unstable than the average for the total transcript population. At high growth rates, mRNAs from categories [G] Carbohydrate and [F] Nucleotide transport and metabolism were more stable than the average for the other transcripts, whereas mRNAs from category [E] Amino acid transport and metabolism had shorter half-lives. These findings are entirely consistent with a previous study highlighting the contribution of changes in mRNA stability at high growth rates to control of the expression of genes involved in the central pathways of carbohydrate and amino acid metabolism [1]. The regulation of mRNA stability varies with gene function, and, for the functional categories described above, with growth rate. The transcripts encoding outer membrane lipoproteins were more stable than the other transcripts only at μ = 0.10 and 0.20 h^−1^. This finding is difficult to interpret.

**Figure 4:**
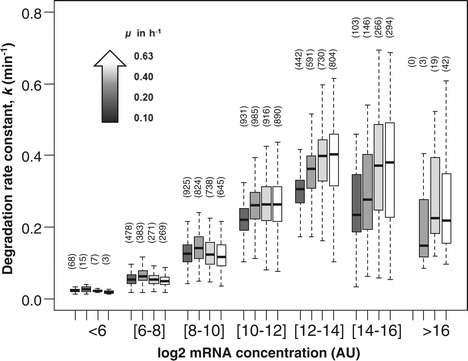
**Effect of growth rate on the relationship between the degradation rate constant** ***k*** **and mRNA concentration.** Boxplot distributions of the degradation rate constant *k* as a function of log2 mRNA concentration (in arbitrary units) are shown for the four growth rates *μ* = 0.10, 0.20, 0.40 and 0.63 h^−1^ (indicated as a black to white color gradient). The numbers of mRNAs in the different box plots are indicated in brackets above each box.

In each of the four models corresponding to different growth rates, mRNA concentration was the factor with the strongest influence but its coefficient decreased slightly with increasing growth rate, suggesting a smaller influence of mRNA levels on mRNA half-life at higher growth rates. We investigated the direct correlation between mRNA concentration and stability in more detail, by plotting the degradation rate constant *k* (ln(2)/t_1/2_) as a function of concentration for all transcripts, at the four growth rates (Figure 4). The data were indeed found to be more dispersed at higher growth rates. Nevertheless, for a given mRNA concentration, the rate of degradation generally increased with growth rate. Thus, independently of mRNA levels, cells were more able to degrade mRNA at higher growth rates. This may reflect the involvement of other factors, such as an increase in the expression of the degradation machinery with increasing growth rate [1]. It may also account for the weaker influence of mRNA concentration on mRNA stability at high growth rates.

### Relationship between mRNA stability and protein level

We investigated the influence of mRNA stability on protein levels in *E. coli*, by carrying out proteomic analyses for the different growth rates (the levels of 643 proteins were quantified, Additional file 2: Table S1) and using new linear covariance models to account for the proteomic results. In addition to the parameters used in the models for half-life, we considered mRNA half-life, the grand average of hydropathicity (GRAVY score, [26]) and the percentages of each amino acid in the protein as explanatory variables. As mRNA concentration and stability were strongly correlated, we did not include mRNA concentration in the models.

The adjusted R^2^ values for protein concentration determination models including mRNA half-life data were about 0.50, and were similar to those of models excluding mRNA half-life data (data not shown). The correlation between mRNA stability and protein level was therefore weak. However, in models including mRNA half-life data, this parameter was selected on the basis of the AIC at μ = 0.10 h^−1^ and μ = 0.63 h^−1^ (Table 2). The estimated coefficients were significant but small, with opposite signs at the two growth rates (−0.14, and +0.08 at μ = 0.10 and 0.63 h^−1^, respectively). These data suggest that the weak correlation between mRNA half-life and protein level may be dependent on growth rate. The low impact of mRNA stability on protein level and the sign inversion at high growth rate were quite surprising because (i) protein level and mRNA concentration were strongly positively correlated for 643 mRNA and protein pairs in this dataset (estimated model coefficient > 0.50, *P*-value < 10^−55^) and (ii) mRNA concentration and half-life were strongly negatively correlated (as demonstrated above in the mRNA half-life determination models for a population of 1589 mRNAs). This intriguing finding may be accounted for by the 643 mRNAs used in the protein determination models being not entirely representative of the larger set of 1589 mRNAs used in the mRNA half-life determination models, as shown by the non-overlapping densities of mRNA levels for the two datasets (Additional file 3: Figure S2). Nevertheless, our models confirmed the significant positive effect of CAI on protein level (estimated coefficient > 0.48, *P*-value < 10^−31^) (Table 2) [27]. Indeed, CAI, which is related to translation efficiency, has frequently been shown to have a strong positive influence on protein concentration [19-22].

**Table 2:** **Estimated coefficients and** ***P*** **-values of protein level models for a selection of explanatory variables**

**Parameter**	**Dependent variable: protein concentration**							
	***μ*** **= 0.10 h** ^**−1**^		***μ*** **= 0.20 h** ^**−1**^		***μ*** **= 0.40 h** ^**−1**^		***μ*** **= 0.63 h** ^**−1**^	
	**Est. Coeff**	***P-value***	**Est. Coeff.**	***P-value***	**Est. Coeff.**	***P-value***	**Est. Coeff.**	***P-value***
**Half-life**	**−0.14**	*1.7E* ^*−05*^	N.S		N.S		**0.08**	*1.1E* ^*−02*^
**CAI**	**0.48**	*2.4E* ^*−30*^	**0.50**	*3.1E* ^*−37*^	**0.56**	*2.6E* ^*−46*^	**0.54**	*5.4E* ^*−43*^
**Adjusted R** ^**2**^	**0.46**		**0.47**		**0.49**		**0.50**	

The coefficients were estimated by the minimization of least squares.

CAI: Codon adaptation index. N.S means that the parameter was not selected by the AIC algorithm. Model determinants (*P*-value ≤ 0.05) are displayed in bold.

## Discussion

We used the largest and most recent mRNA half-life datasets for *E. coli* and a large number of parameters in linear covariance models, to identify and rank the general parameters associated with mRNA stability at four different growth rates. The determination coefficients (R^2^) of the four models corresponding to the different growth rates were high (>0.51), indicating that a large proportion of mRNA stability could be explained by a combination of the selected general parameters. This finding highlights the importance of general mechanisms for the regulation of mRNA stability. The general parameters correlated with mRNA lifetime can be grouped according to the regulatory mechanisms involved.

The first group of parameters associated with mRNA stability includes parameters directly related to the activity of the degradation machinery and, particularly, RNase E activity. We found that mRNA concentration was the main indicator of mRNA half-life and was negatively correlated with mRNA lifetime. The most concentrated mRNAs are therefore the least stable, consistent with a previous suggestion that mRNA degradation generally tends to counterbalance transcription [1]. This counterintuitive hypothesis has also been put forward for other bacteria [13,16]. It can be explained by high mRNA concentrations leading to a higher probability of encountering RNases, resulting in a shorter half-life of the mRNA. Furthermore, we showed, by analyzing the degradation rate constant as a function of growth rate, that the ability of cells to degrade mRNA increased with increasing growth rate. This is consistent with the hypothesis proposed above, because expression of the degradation machinery is generally upregulated and total mRNA concentrations are higher at high growth rates [1]. Generally, mRNA levels need to be compatible with the constraints of the growth conditions. The rapid turnover of highly expressed messengers is probably required for the rapid adaptation of cell physiology in a changing environment.

The weak but significant negative correlation between ORF length and mRNA half-life is consistent with the widely held view that endonucleolytic attack by RNase E is the rate-limiting step in mRNA degradation and that the cleavage sites used in the alternative direct entry pathway are evenly distributed along the length of the transcript [28]. However, we cannot rule out the possibility that mechanical damage renders long mRNAs less stable than short ones [17]. Furthermore, we showed that, for most polycistronic mRNAs, a greater distance of the cistron from the 5′-end is associated with higher stability, consistent with the findings of Selinger and coworkers [29]. However, we also provide the first demonstration that this phenomenon is independent of growth rate. These results are entirely consistent with the current model of 5′ to 3′ mRNA degradation initiated via the 5′ end-dependent RNase E endonucleolytic pathway [28,30].

Studies with a model RNA showed that RNase E had a preference for AU-rich regions [7]. However, an association of AU-rich motifs and GC content in the 5′UTR with mRNA stability has never been definitively demonstrated at the whole-genome level [9]. Our linear models show that there is a negative correlation between GC content and half-life values, but only for the complete 5′UTR + ORF sequence, not for the 5′UTR or ORF considered alone. We therefore confirm the absence, at genome-wide level, of a correlation between the GC content of the 5′UTR and transcript stability. Our results differ slightly from those of Lenz and coworkers, who described a negative correlation between GC content and mRNA half-life, but only for the ORF [31]. These authors showed that the correlation between GC content and half-life values was not simple, instead varying along the length of the mRNA molecule. This may explain why this parameter was not identified as having an influence on mRNA stability. Given that the formation of secondary structures is often linked to GC content, it is not surprising that the *Z* score for the folding energy of the −30 / +24 bp region of the mRNA was not identified as a parameter affecting mRNA stability in any of the models. We also suggest that the G/C-rich motif CUGGC, located in the 5′UTR region, stabilizes mRNAs at low growth rates.

The second group of parameters associated with mRNA stability includes parameters directly related to ribosome activity and, thus, translation. Surprisingly, we found that CAI was positively correlated with mRNA half-life. This observation is not consistent with the simple negative correlation of tRNA adaptation index (tAI) and half-life reported for *E. coli* [31] but is consistent with results for *L. lactis* [13]. A high CAI is believed to reduce ribosome pausing, thereby leading to efficient elongation of the translation product [32,33]. Given the well known protective effect of ribosomes against RNase attacks [34], we suggest that efficient translation elongation, leading to the continuous, rapid movement of the ribosome along the mRNA, is likely to impede the interaction of nucleases with their substrate, thereby protecting mRNA against degradation. A second hypothesis potentially accounting for these findings makes use of the opposite idea: a deleterious effect of ribosomes on mRNA stability due to their ability to recruit the RNA degradosome [35]. In the presence of a high CAI, rapid translation would result in only a small number of ribosomes binding to each messenger. This would lead to fewer degradosome complexes being recruited per mRNA molecule, thereby increasing mRNA stability. We also observed that the AGGAG motif located in the ribosome binding site was associated with greater mRNA stability. This supports the hypothesis of a protective effect of ribosomes against mRNA degradation.

Other parameters related to mRNA stability were identified in this study, but the mechanisms by which these parameters affect mRNA half-life are unknown. These new parameters were linked to gene functions probably dependent on cell physiology requirements and bacterial adaptation. Transcripts from functional categories relating to growth, such as categories [D] Cell cycle control, cell division, chromosome partitioning and [J] Translation, ribosomal structure and biogenesis, were significantly more stable than the average at all growth rates. As mRNA stability determines the time for which a transcript is accessible for translation, the rate of decay of these growth-related mRNAs is probably kept low, to ensure that basal levels of these mRNAs are present. This hypothesis is supported by our demonstration that essential genes have significantly longer mRNA half-lives than other genes. Similarly, the stability of the cistrons of long operons was greater than that of the cistrons of shorter operons. Given that operons are often considered to be functional structures, the stability of these cistrons is probably programmed to satisfy the requirements for the functions of the products they encode, as demonstrated in yeast [10]. Moreover, the half-lives of mRNAs for some functional categories were specifically regulated at certain growth rates. For example, the stabilization of transcripts from functional categories such as [G] Carbohydrate and [F] Nucleotide transport and metabolism at the highest growth rate may support higher rates of metabolism [1]. Taken together, these data suggest that the functional genomics findings probably reflect the specific regulation of mRNA stability according to the role of the gene concerned in cell physiology and adaptation.

Our linear covariance models had high determination coefficients, but the values obtained were always lower than 1. This indicates that other factors not investigated here are also involved in mRNA half-life determination. Furthermore, as R^2^ decreased with increasing growth rate, these uninvestigated parameters made a greater contribution at higher growth rates. These parameters remain to be identified, but they may be related to RNA-binding proteins, riboswitches and sRNAs with growth rate-dependent effects [36,37].

Finally, we showed that the contribution, at the genome-wide scale, of mRNA stability to protein levels was small, whatever the growth rate considered. A significant but small correlation between mRNA half-life and protein abundance was previously reported in *E. coli* [31]. This result highlights the complexity of protein level regulation, which involves other complex and highly regulated processes, such as translation and protein turnover, in addition to mRNA stability and transcription.

## Conclusions

In this work, we identified general parameters associated with mRNA stability, using half-life measurements obtained for all the mRNAs of *E. coli* at different growth rates. We provide, for the first time in *E. coli*, a ranking of these parameters in terms of their level of influence on mRNA stability. This study made it possible to generalize certain features previously correlated with stability for particular mRNAs, such as ORF length and translatability, to the entire population of transcripts, thus demonstrating their importance. However, such generalization was not possible for all the expected parameters and we also identified new parameters influencing mRNA stability that were not anticipated. In particular, mRNA concentration was identified as the main factor correlated with mRNA stability. There was an unexpected strong negative correlation between mRNA concentration and stability (especially at low growth rates), indicating that more abundant mRNAs are degraded more rapidly. It would now be interesting to apply this genome-wide analysis of transcript lifetimes systematically to other microorganisms (e.g. yeast, *Bacillus*) to determine the organism-specificity of mRNA stability indicators and to address the question of the differences in mRNA degradation mechanisms between microorganisms.

## Methods

### Data compilation

We obtained mRNA half-life and expression data for 2947 genes, for *E. coli* growing at four different rates (μ = 0.10, 0.20, 0.40 and 0.63 h^−1^), from a previous study [1]. ORF lengths and sequences were retrieved from the Ecocyc database (http://www.ecocyc.org/). The 5′UTR lengths and sequences of all the mRNAs considered were experimentally determined in a previous study [38]. If several alternative transcription start sites (TSS) had been identified for a transcript, we used the TSS furthest from the site of translation initiation in the analysis, to minimize the loss of sequence information. The second nucleotide (purine/pyrimidine) of each mRNA was identified for these 5′UTR sequences. We obtained 5′UTR + ORF sequences and lengths by combining the results for the 5′UTR and ORF described above. We retrieved transcription units from the RegulonDB database (http://regulondb.ccg.unam.mx/), to determine the number of genes in operons and their positions within the polycistronic mRNA. Clusters of orthologous groups of protein (COG) annotations and the cellular distributions of gene products were obtained from the *E. coli* K-12 annotation [39]. Essential genes were identified by determining knockout efficiency, as previously described [23]. The compiled data are presented in Additional file 4: Table S2.

### Sequence-related features of mRNA and proteins

The GC contents of the 5′UTR and ORF were calculated as the percentage of G and C nucleotides in each sequence. Peptide signals in ORF sequences were predicted with SignalP4.1 software (http://www.cbs.dtu.dk/services/SignalP/). The codon adaptation index (CAI) of the ORF was calculated as previously described [18], with 55 ribosomal proteins used as a reference. We calculated the *Z* score as a measurement of thermodynamic stability for the −30 to +24 bp region of each mRNA relative to its start codon. The *Z* score was defined for each region as the minimum free folding energy of the native sequence minus the mean minimum free folding energy of its randomized sequences divided by the standard deviation of the minimum free folding energy of its randomized sequences. Randomized sequences were computed by shuffling each region of interest 100 times, with Ushuffle software [40] which conserves dinucleotide composition. The minimum free folding energy of native and randomized sequences was estimated with the RNAfold module of the Vienna Package [41]. The grand average of hydropathicity (GRAVY) score for the linear polypeptide sequence was calculated as the sum of hydropathy values for all amino acids, divided by the number of residues in the sequence [26].

### Determination of protein levels

Cells from steady-state continuous cultures of *E. coli* growing at four different rates [1] were centrifuged and the cell pellets were washed with ice-cold PBS buffer and centrifuged again. The cell pellets were frozen in liquid nitrogen and stored at −80°C. They were then resuspended in lysis buffer (4% SDS, 100 mM Tris–HCl pH 8, 100 mM DTT), heated for 5 minutes at 95°C and cleared by sonication. Total protein concentration was then determined by measuring tryptophan absorbance. A SILAC (for stable isotope labeling by amino acids in cell culture) approach [42] was used for the relative quantification of proteins for the four growth rates. Absolute protein quantification was then performed by the iBAQ (intensity-based absolute quantification) method, with the addition of UPS2 proteomic standards (Sigma-Aldrich) to the samples [43].

### Linear covariance models

A linear analysis of covariance model was used to identify the major determinants of mRNA stability at the four growth rates; various quantitative and qualitative parameters were included in this model. The parameters included in the model included gene parameters, such as mRNA concentration (*[mRNA]*), length (*ORF length, 5′UTR length, 5′UTR + ORF length*) and GC content (*%GC ORF, %GC 5′UTR* and *%GC 5′UTR + ORF,* respectively), CAI and *Z* score for folding energy (*Zscore*) were considered as quantitative parameters. They were log-transformed to obtain a normal distribution (except for *Z* score, which is, by definition, normally distributed) and all were centered and reduced. This normalization was applied to allow an adjustment of their levels and a comparison of model coefficients. The number of genes in the operon containing the gene considered (*No. of genes in operon*), gene essentiality (*Essential*), COG membership, the cellular distribution of the gene product, the presence of a peptide signal in the mRNA and the nature of the second nucleotide in the mRNA (*Nature of 2nd nt*) were considered as qualitative parameters. Equation (1) describes the models established to explain the mRNA half-lives at a given growth rate. We established one model for each of the growth rates: 0.10, 0.20, 0.40 and 0.63 h^−1^.

1 $$ \begin{array}{c} \ln \left( mRNA\  stabilit{y}_{\left(i,j\right)}\right)=\alpha +{\beta}_{{\left[ mRNA\right]}_{\left(i,j\right)}} \ln \left({\left[ mRNA\right]}_{\left(i,j\right)}\right)+{\beta}_{ORF\ \mathit{\ell engt}{h}_{\left(\mathrm{i}\right)}} \ln \left( ORF\  lengt{h}_{(i)}\right)\\ {}+{\beta}_{5^{\hbox{'}}UTR\  lengt{h}_{\left(\mathrm{i}\right)}}\  \ln \left({5}^{\mathit{\hbox{'}}}UTR\  lengt{h}_{(i)}\right)+{\beta}_{5^{\hbox{'}}UTR+ ORF\  lengt{h}_{\left(\mathrm{i}\right)}} \ln \left({5}^{\hbox{'}}UTR+ OR F\  lengt{h}_{(i)}\right)\ \\ {}+{\beta}_{\%GC\  OR{F}_{(i)}} \ln \left(\%GC\  OR{F}_{(i)}\right)+{\beta}_{{}_{\%GC\ 5\hbox{'}UT{R}_{\left(\mathrm{i}\right)}}} \ln \left(\%GC\  5\mathit{\hbox{'}}UT{R}_{(i)}\right)\kern0.75em \\ {}+{\beta}_{\%GC\ {5}^{\hbox{'}}UTR+ OR{F}_{\left(\mathrm{i}\right)}} \ln \left(\%GC\ {5}^{\mathit{\hbox{'}}}UTR+ OR{F}_{(i)}\right)+{\beta}_{CA{I}_{\left(\mathrm{i}\right)}} \ln \left(CA{I}_{(i)}\right)+{\beta}_{Zscor{e}_{\left(\mathrm{i}\right)}} \ln \left( Zscor{e}_{(i)}\right)\\ {}+{\lambda}_{No. of\  genes\ \mathrm{in}\  opero{n}_{\left(\mathrm{i}\right)}}+{\lambda}_{Essentia{l}_{\left(\mathrm{i}\right)}}+{\lambda_{COG}}_{{}_{\left(\mathrm{i}\right)}}+{\lambda}_{Ce\mathit{\ell \ell }\ \mathit{\ell ocatio}{n}_{\left(\mathrm{i}\right)}}+{\lambda}_{Signal\  peptid{e}_{\left(\mathrm{i}\right)}}\\ {}+{\lambda}_{Nature\  of2ndn{t}_{\left(\mathrm{i}\right)}}+{\varepsilon}_{(i)}\kern2.5em \end{array} $$

where *mRNA stability(i,j)* is the measured level of the *i*th value for the variable of interest, *j* is the *j*th growth rate, *α* is the intercept, *β* and *λ* are the coefficients associated with the quantitative and qualitative parameters, respectively, and *ε*_*(i)*_ is the error term for the *i*th half-life. A complete matrix (without missing values) for all the parameters and half-lives at the four growth rates was obtained for 1589 messengers. We then used the Akaike information criterion (AIC) to select the model for which the compromise between fitting quality and complexity was best. This model selection process is derived from the classical least-squares minimization method used to estimate model coefficients, but with an additional penalization term that increases with the complexity of the model [44]. The Akaike selection procedure is designed to identify the model minimizing the following criterion:

$$ \mathrm{n}\  \log \left(\mathrm{R}\mathrm{S}\mathrm{S}/\mathrm{n}\right)+2\mathrm{N} $$

where *n* is the number of mRNA stabilities used to construct the model and N denotes the number of coefficients estimated by the model. This approach makes it possible to select the most significant parameters without the need for *a priori* subjective selection, which might bias the results. The coefficients of the qualitative parameters cannot be compared with those of the quantitative parameters. The coefficients of the quantitative parameters influence the slope of the line describing the model, whereas those of the qualitative parameters modify the intercept. The influence of each quantitative parameter can thus be estimated by ranking the absolute values of the associated coefficients. The coefficients of qualitative parameters must be interpreted differently. For these coefficients, a positive value is associated with a half-life of the corresponding mRNA longer than the overall mean value. Conversely, a negative value is associated with below-average transcript stability. A *P*-value was calculated for each parameter coefficient. The AIC selection procedure (stepAIC function) and estimation of the regression and determination coefficients (lm function) of the linear models were performed with R-free statistical software (http://www.r-project.org/).

### Search for sequence motifs

We included in the analysis the 1937 5′UTR sequences experimentally determined [38] of mRNAs with a measured half-life value (this study). When the 5′UTR sequence was longer than 100 nucleotides, we limited the motif search to the 100-nucleotide region immediately upstream from the initiation codon. We searched for stabilizing and destabilizing sequence motifs in the top (most stable) and bottom (least stable) quartiles of the mRNA half-life distribution. Eight datasets were constructed, each containing the 485 most or least stable mRNAs, separately, for μ = 0.10, 0.20, 0.40 and 0.63 h^−1^. We used RMES software (http://migale.jouy.inra.fr/?q=rmes, [24]) to search for differences between unstable and stable mRNAs in terms of the motifs present in the 5′UTR sequences for these subgroups. We assessed the over- and underrepresentation of five-nucleotide motifs in these sequences, with a first-order Markov Model (M1) and a compound Poisson approximation. RMES was used to calculate *P*-values and scores for all 1024 possible five-nucleotide motifs, by comparing the observed number of times each motif occurred with the number expected for random sequences of the same composition in terms of two-nucleotide “words”. The *P*-values obtained correspond to the probability, under our model, of observing as many, or as few occurrences of the motif as were actually observed. RMES scores are derived from *P*-values by standard one-to-one probit transformation and can be used to distinguish between exceptionally frequent motifs (with high positive scores) and exceptionally rare motifs (with high negative scores).

### Availability of supporting data

All the supporting data are included as additional files.

## Additional files

Additional file 1: Figure S1. Half-life densities of mRNAs with low *Z* scores (< −2) (in black) and the rest of the mRNA population (in red) at different growth rates. Additional file 2: Table S1. Protein concentrations at four growth rates. Additional file 3: Figure S2. Density curves for mRNA levels. The density curve for the mRNA levels of the 643 mRNAs included in the protein level model is shown in blue. The density curve of the mRNA levels for the 1589 mRNAs included in the half-life model is shown in red. Density curves are shown for each of the growth rates studied. Additional file 4: Table S2. Compilation of data used as inputs for the modeling of mRNA half-life.
