# Supplementary figures and images for: Genome-wide investigation of mRNA lifetime determinants in Escherichia coli cells cultured at different growth rates
Source: BMC Genomics. 2015 Apr 9;16(1):275. doi: 10.1186/s12864-015-1482-8 (PMC4421995; doi:10.1186/s12864-015-1482-8)

## Slide 1
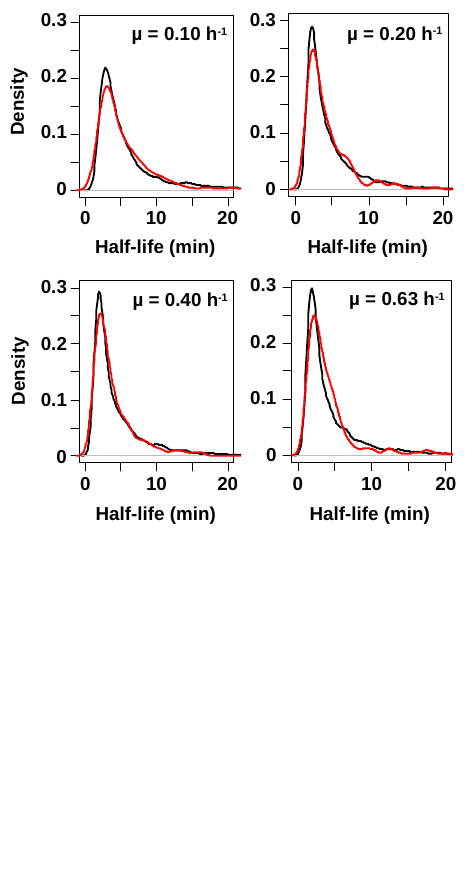

0.3
0.3
µ = 0.20 h-1
µ = 0.10 h-1
0.2
0.2
Density
0.1
0.1
0
0
0
10
20
0
10
20
Half-life (min)
Half-life (min)
0.3
0.3
µ = 0.63 h-1
µ = 0.40 h-1
0.2
0.2
Density
0.1
0.1
0
0
0
10
20
0
10
20
Half-life (min)
Half-life (min)

Supplement: Additional file 1: Figure S1. — Half-life densities of mRNAs with low Z scores (< −2) (in black) and the rest of the mRNA population (in red) at different growth rates. [file 12864_2015_1482_MOESM1_ESM.pptx]

## Slide 1
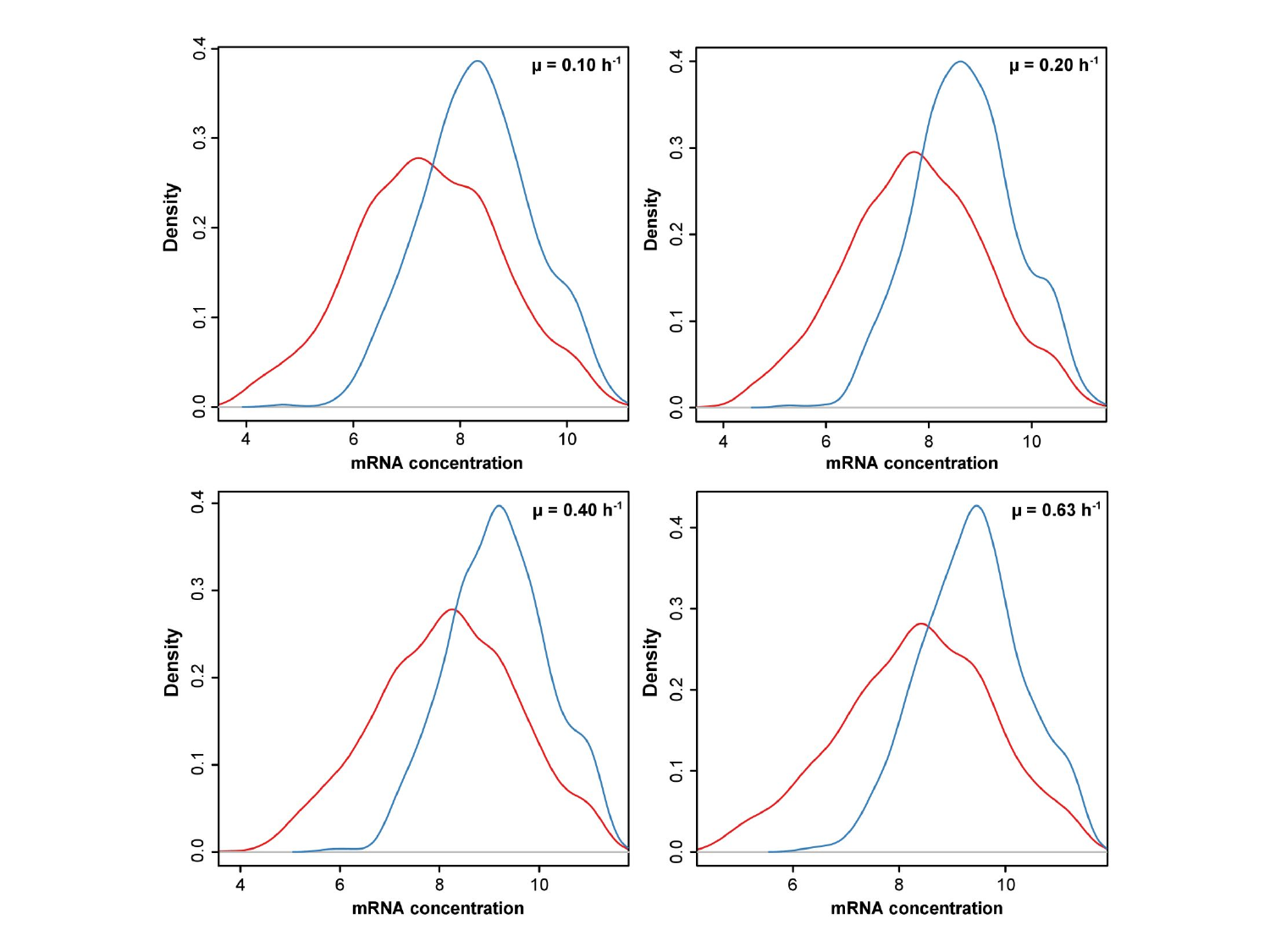

Supplement: Additional file 3: Figure S2. — Density curves for mRNA levels. The density curve for the mRNA levels of the 643 mRNAs included in the protein level model is shown in blue. The density curve of the mRNA levels for the 1589 mRNAs included in the half-life model is shown in red. Density curves are shown for each of the growth rates studied. [file 12864_2015_1482_MOESM3_ESM.pptx]
